# Impact of the Precursor on the Physicochemical Properties and Photoactivity of TiO_2_ Nanoparticles Produced in Supercritical CO_2_

**DOI:** 10.3390/nano13162328

**Published:** 2023-08-13

**Authors:** Óscar Ramiro Andrade, Rafael Camarillo, Fabiola Martínez, Carlos Jiménez, Jesusa Rincón

**Affiliations:** Department of Chemical Engineering, Faculty of Environmental Sciences and Biochemistry, University of Castilla-La Mancha, 45071 Toledo, Spain

**Keywords:** TiO_2_, supercritical CO_2_, semiconductor nanoparticles synthesis, precursor, CO_2_ photoreduction

## Abstract

The synthesis of TiO_2_ nanoparticles (NPs) in supercritical media has been reported over the last two decades. However, very few studies have compared the physicochemical characteristics and photoactivity of the TiO_2_ powders produced from different precursors, and even fewer have investigated the effect of using different ratios of hydrolytic agent/precursor (HA/P) on the properties of the semiconductor. To bridge this knowledge gap, this research focuses on the synthesis and characterization of TiO_2_ NPs obtained in a supercritical CO_2_ medium from four different TiO_2_ precursors, namely diisopropoxytitanium bis (acetylacetonate) (TDB), titanium (IV) isopropoxide (TIP), titanium (IV) butoxide (TBO), and titanium (IV) 2-ethylhexyloxide (TEO). Further, the effect of various HA/P ratios (10, 20, 30, and 40 mol/mol) when using ethanol as a hydrolytic agent has also been analyzed. Results obtained have shown that the physicochemical properties of the catalysts are not significantly affected by these variables, although some differences do exist between the synthesized materials and their catalytic performances. Specifically, photocatalysts obtained from TIP and TEO at the higher HA/P ratios (HA/P = 30 and HA/P = 40) led to higher CO_2_ photoconversions (6.3–7 µmol·g^−1^·h^−1^, Apparent Quantum Efficiency < 0.1%), about three times higher than those attained with commercial TiO_2_ P-25. These results have been imputed to the fact that these catalysts exhibit appropriate values of crystal size, surface area, light absorption, and charge transfer properties.

## 1. Introduction

Titanium dioxide is a versatile material that has captured the attention of researchers in many fields, both in academic and industrial circles. In particular, in the area of environmental remediation, TiO_2_ has been successfully applied for the photocatalytic degradation of organic pollutants from water and air [1,2], its outstanding performance being attributed to both excellent optical and electronic properties and high chemical stability [3]. Other highly appreciated features of TiO_2_ as a photocatalyst are its low cost, non-toxicity, and eco-friendliness [4].

Titanium dioxide can be prepared using different methodologies. Those most reported in the literature include sol-gel, solvothermal/hydrothermal, sonochemical, and physical and chemical vapor depositions [5]. Particularly, the solvothermal technique, which involves the crystallization of TiO_2_ from a non-aqueous solution of the metal precursor under controlled temperature and pressure, is very effective [6]. Further advantages of this method are its low cost and that it allows obtaining a high yield of pure nanoparticles with high crystallinity and good size control [7]. Moreover, a greener TiO_2_ synthesis process may result if supercritical CO_2_ is used as a substitute for traditionally used organic solvents (toluene, benzene, etc.) [8].

Accordingly, various investigations have examined this supercritical solvothermal synthesis approach [9,10,11,12,13,14,15,16,17]. For example, Reverchon et al. [16] used a CO_2_/water supercritical medium for the synthesis of titanium hydroxide nanoparticles from titanium tetraisopropoxide (TIP), with water taking part in the reaction as a hydrolytic agent. Reaction media CO_2_/ethanol and CO_2_/isopropanol were selected by Alonso et al. [14,15] for the synthesis of TiO_2_ from two different precursors: TIP and diisopropoxytitanium bis (acetylacetonate) (TDB). These same precursors were also employed by our own group to produce nanoparticles and nanofibers of bare TiO_2_, metal- and non-metal-doped TiO_2_, and TiO_2_-based hybrid photocatalysts [9,10,11,12,13].

From these former studies, it was concluded that TiO_2_ properties varied depending on the synthesis conditions in which nanoparticles were formed. Specifically, Alonso et al. [14,15] reported that TiO_2_ parameters such as crystallinity, surface area, particle morphology, particle size, chemical purity, and photoactivity were related to operational variables such as temperature, pressure, addition of cosolvent, and precursor concentration. These results were confirmed and expanded in later works [1,10,13]. Additionally, Camarillo et al. [12] showed that the calcination temperature of the TiO_2_ powders produced in a supercritical medium was decisive for obtaining the anatase or rutile crystalline phase of TiO_2_. However, neither the effect of the TiO_2_ precursor nor the hydrolytic agent/precursor ratio used for the TiO_2_ synthesis on the physicochemical and photocatalytic properties of the semiconductor have been thoroughly analyzed yet.

Therefore, considering (1) that the photocatalytic activity of TiO_2_ is influenced by its physicochemical properties [3], such as surface area, particle size and morphology, porosity, crystal structure, surface hydroxyl group density, etc., (2) that all these structural parameters are dependent on the TiO_2_ synthesis process [18], such as synthesis pressure and temperature, kind of precursor or hydrolysis reactant used, etc., and (3) that the effect of TiO_2_ precursor and hydrolytic agent/precursor ratio on TiO_2_ properties still remains unsolved; in this study, the physicochemical properties and photoactivity of TiO_2_ nanoparticles produced from four different precursors (TDB, TIP, TBO (acronym for titanium (IV) butoxide) and TEO (titanium (IV) 2-ethylhexyloxide)) in supercritical CO_2_/ethanol medium and using four hydrolytic agent/precursor ratios (10, 20, 30, and 40 mol/mol) are thoroughly investigated. These precursors and hydrolytic agent/precursor ratios were selected attending to those most frequently used in earlier studies of the solvothermal supercritical synthesis of TiO_2_ [12,16,19,20].

Regarding the techniques used for the characterization of the synthesized materials, they were X-ray diffraction (XRD), Fourier transform infrared spectroscopy (FT-IR), transmission electron microscopy (TEM), scanning electron microscopy (SEM), UV–vis diffuse reflectance spectroscopy (UV-vis DRS), electrochemical impedance spectroscopy (EIS), and the BET method. As for the analysis of the TiO_2_ photoactivity, the CO_2_ photoreduction has been employed as a photocatalytic reaction model due to the interest it arouses in the fields of energy and environment; namely, it allows the recycling of CO_2_ from fossil sources to new fuel molecules.

In summary, the aim of this work has been to establish the effect of two operational synthesis conditions (type of precursor and hydrolytic agent/precursor ratio) on the TiO_2_ physicochemical properties and photoactivity. It is a really important issue because the clear understanding of the relationship between the operational variables that control the formation of TiO_2_ and its subsequent properties is a problem that must be solved in order to successfully address the fine tuning of the photocatalyst properties required for a given application.

## 2. Methodology

### 2.1. Chemicals

The following reagents were used as titania precursors: diisopropoxytitanium bis (acetylacetonate) solution, purum 75% in isopropyl alcohol (TDB), titanium (IV) butoxide 97% (TBO), and titanium (IV) 2-ethylhexyloxide 95% (TEO), all of them provided by ALDRICH (Darmstadt, Germany). Also, titanium (IV) isopropoxide 98% (TIP) was acquired from ACROS (Madrid, Spain). Ethanol absolute from Scharlab (Barcelona, Spain) was employed as a hydrolytic agent and cosolvent.

### 2.2. TiO_2_ Synthesis

**Figure 1:**
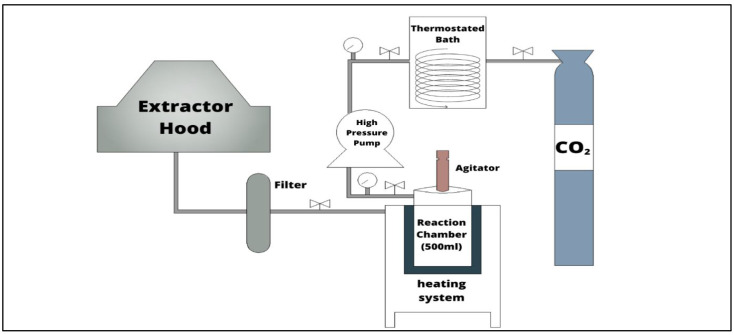
Representation of the SC synthesis set-up.

First of all, both titanium precursor (P) and hydrolytic agent (HA) were placed inside the reactor (ILSHIN, Daejeon, South Korea, RS500-SMH, volume 500 mL) (Figure 1). Subsequently, CO_2_ was introduced into the reactor by means of a high-pressure pump (Thar SFC, Pittsburgh, PA, USA, series P), previously cooled by passing through a thermostatic bath (Selecta, Barcelona, Spain, Frigiterm-30). After that, the reactor was programmed to reach the desired temperature (250 °C), with continuous stirring (300 RPM) for the specified reaction time (2 h) and pressure (20 MPa). More details about this process are described in a previous work [10]. Four precursors were used in the syntheses: TDB, TBO, TEO, and TIP. The variations of the HA/P ratios were established at 10, 20, 30, and 40 mol/mol.

Once the synthesis process was finished, the TiO_2_ nanoparticles (NPs) were removed from the reactor installation. Next, the NPs produced were dried. The drying process, which is required prior to their calcination, was carried out at 105 °C for a period of 12 h. The dried catalysts were then calcinated at 400 °C for a period of 6 h to remove C pollution [21]. The calcination temperature was selected considering the transition temperature between anatase and rutile phases, thus avoiding an excessive amount of rutile, which takes place at higher temperatures [13,22].

### 2.3. TiO_2_ Characterization

All TiO_2_ NPs were characterized using several analytical techniques. To simplify their identification, they were coded attending to the ratio between the moles of hydrolytic agent (HA) and precursor (P) used in the catalyst synthesis, as shown in Table 1.

An X-ray powder diffractometer (XRD, Philips X′Pert MPD, Amsterdam, The Netherlands) was utilized to determine crystallinity and crystalline phase of the catalysts. Using the Scherrer equation, crystallite size was calculated [23]. A Fourier transform infrared transmission spectroscopy (FTIR) analysis was executed to determine the presence of surface functional groups on the composites’ surface. FTIR was performed with a Perkin-Elmer Spectrum 100 FTIR spectroscope (Madrid, Spain) equipped with MIR DTGS (Mid-IR Deuterated Triglycine Sulphate) and MCT (Mercury Cadmium Telluride). For electrochemical impedance spectroscopy (EIS), a potentiostat (AUTOLAB PGSTAT302N, Utrecht, The Netherlands), a 0.1 M KHCO_3_ solution as electrolyte, a calomel electrode as reference electrode, a Pt electrode as a counter electrode, and a frequency range 1–100,000 Hz [9] were used. To support the catalyst for EIS measurement, the TiO_2_ NPs were dispersed in 2-propanol to make a catalytic ink, and with the help of an airbrush, the catalytic ink was sprayed on a Carbon Paper 90 (Toray TGP-H-090, Tokyo, Japan). The specific surface area of the powders was measured using a BET area analyzer (Quantachrome Nova Touch LX2, Graz, Austria). Finally, the absorption thresholds and band gap energies were obtained with a diffuse reflectance (DR) UV–vis spectrophotometer (Jasco, Croissy-sur-Seine, France, V650) by means of a graphical method previously described [23].

### 2.4. Photocatalytic Reaction Test

To determine the photocatalytic activity of the TiO_2_ NPs, the photoreduction of CO_2_ was used as a model of photocatalytic transformation. To this end, an experimental installation previously described [2,13] was used. First, catalysts were fixated to a filter, which later was placed in the reaction chamber. With the reaction chamber closed, a cleaning procedure with helium was performed. Once the equipment had been cleaned and humidified to the required humidity level, CO_2_ was introduced into the chamber and a lamp that simulated sunlight was turned on. Then, after the reaction time was over (3 h), the obtained gas mixture was analyzed using a gas chromatograph (Agilent Technologies, Santa Clara, CA, USA, 490 Micro GC).

## 3. Results

### 3.1. Synthesis Yield

After the synthesis and drying processes of the catalysts, the only difference observable with the naked eye was the variation in the brown tone of the recovered material. Catalysts from TDB appeared in the form of granular particles with a shade of brown close to the orange tones, those from TIP and TBO were, respectively, light brown and dark brown, and, finally, TEO catalysts showed a brighter but still dark brown shade. However, after the calcination process, all the catalysts turned into a grayish white powder, almost with no variation between precursors.

**Figure 2:**
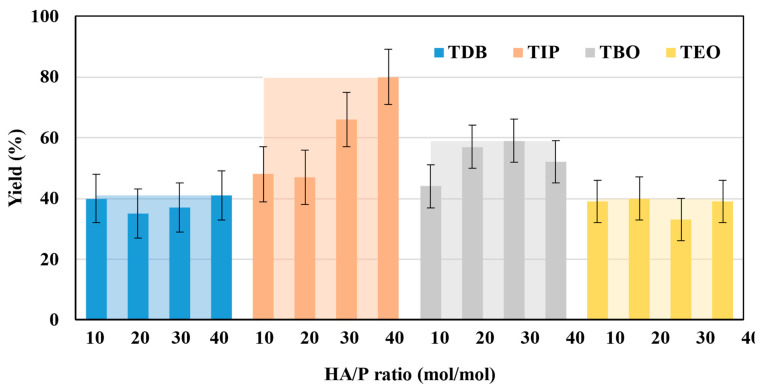
Production yield of TiO_2_ catalysts, with solid color columns representing the HA/P ratio average and the clear areas behind representing the precursor maximum.

Despite the above, precursors and HA/P ratios did affect the yield of the synthesis process. This variable is defined as the ratio between the moles of catalyst produced and the moles of precursor employed, considering that the synthesis reaction involves a 1:1 ratio for all precursors. Results obtained are presented in Figure 2, which shows the differences in yields between precursors and HA/P ratios.

For TIP catalysts, increasing yields are observed as the HA/P ratio increases. Thus, the highest yield (80%) corresponds to the highest HA/P ratio tested (40 mol/mol). It should be noted that this value is also the highest of those obtained in this research. In the case of TDB catalysts, the maximum yield is much lower, close to 40%, and it remains almost constant for all HA/P ratios employed. Finally, the same happens with TBO and TEO, the maximum yields being about 60% and 40%, respectively. As in the case of TDB catalysts, the small variations of yields observed at different HA/P ratios when using these last precursors can be attributed to the experimental error. However, the larger differences found in the case of TIP may be imputed to the higher reactivity of the precursor. In effect, with TIP being more reactive than the other precursors due to its structure, which can be easily hydrolyzed and participate in the subsequent condensation process, it is easier for the HA/P ratio to influence the hydrolysis and condensation reactions and, therefore, the process yield [24,25].

On the other hand, the fact that TDB and TEO are the precursors leading to the lowest yields can also be attributed to their higher thermal and kinetic stabilities because of the complexity of their ligands [15,26]. In particular, as shown in Table 2, TDB has two acetylacetonate ligands and two isopropoxi ligands while TEO has four long alkyl chains of 2-ethylhexyloxide. Therefore, having more complex ligands, hydrolysis of TDB and TEO is expected to be more difficult than for TIP or TBO. In fact, Kinoshita et al. [19] reported a slower hydrolysis rate for TDB and TEO as compared to precursors with simpler ligands. Therefore, the higher production yields obtained in this work with TIP and TBO can be attributed to the fact that their molecules can be more easily hydrolyzed than those of TDB and TEO, even more in the case of TIP, probably due to its lower molecular weight.

Accordingly, considering only the yield, TIP emerges as the best precursor, especially at the HA/P ratio of 40 mol/mol, for which the highest yield was achieved (80%).

Comparing these results with previous studies, it can be mentioned that Alonso et al. [15] reported that at low alcohol/precursor ratios (around 10 mol/mol), a wet pasty fluid was obtained. However, in the present research, similar to Kinoshita et al. [19], TiO_2_ nanoparticles were obtained at this ratio (10 mol/mol). To produce an unrecoverable white pasty fluid, the ratio had to be reduced to 2 mol/mol. It should also be mentioned that Alonso et al. [15] and Camarillo et al. [27] found for TDB and TIP slightly higher yields than those reported here for both precursors when operating at similar synthesis conditions. A possible explanation for this result could be the use of a larger volume reactor (500 mL) compared to the one used in previous works (100 mL). The larger reaction volume makes it possible to obtain a greater quantity of product but, in turn, entails a greater loss of product during its recovery from the reaction vessel.

### 3.2. TiO_2_ Physicochemical Characteristics

#### 3.2.1. SEM/TEM

**Figure 3:**
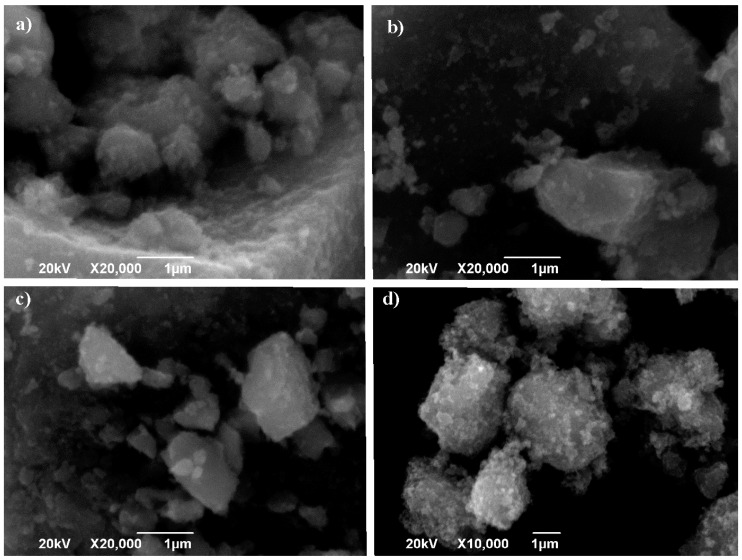
SEM images of selected catalysts: (**a**) TDB-30, (**b**) TIP-30, (**c**) TBO-30, and (**d**) TEO-30.

Considering the results obtained, as they did not seem to show a substantial variation for the different HA/P ratios tested, only one catalyst from each precursor was selected for SEM/TEM analysis. The selected catalysts were the ones synthetized with the HA/P ratio of 30 mol/mol for TDB, TIP, TBO, and TEO. As it can be appreciated in Figure 3, for SEM images, all the catalysts present very small and dispersed particles which agglomerate to form grains or denser structures like blocks with various morphologies and dimensions (1–2 µm). The TiO_2_ crystals for TIP-30 and TBO-30 (Figure 3b,c) appear more in a diverse combination of polyhedral particles that could form a structure similar to a monolithic structure. For TDB-30 and TEO-30 (Figure 3a,d), the particles present a quasi-spherical shape similar to commercial nanoparticles (P-25) but less filamentous [27]. For all the catalysts, the surfaces of the grains appear to be smooth, but at a bigger scale the crystallites seem to be porous [28,29]. The difficulty of controlling particle shape through the traditional solvothermal method has been reported, thus the different shapes and agglomerates are observed for all the precursors. Also, when the synthesis process is developed using supercritical fluids, mixtures of different particle geometries are also observed [19,27].

In comparison with other studies [19,27], which present uniform, spherical, and smooth particles, the nanoparticles obtained with TDB seem less regular in size and morphology (Figure 3a). This fact is not necessarily negative, since some authors associate the mixture of particle shapes with better photoactivity of the catalysts [27]. They also appear more porous than in other studies. For nanoparticles obtained with TIP (Figure 3b), in comparison with previous studies carried out by our group [27], a similar distribution of morphology is noted; however, they present a smoother surface on the agglomerates and the particles. For TBO, Kinoshita et al. [19] report a needle-like and spherical morphologies, but in our case (Figure 3c) the morphology is similar to the one presented by TIP (Figure 3b). In the same study, Kinoshita et al. report nanoparticles obtained with TEO with similar morphology and surface as in this study (Figure 3d).

**Figure 4:**
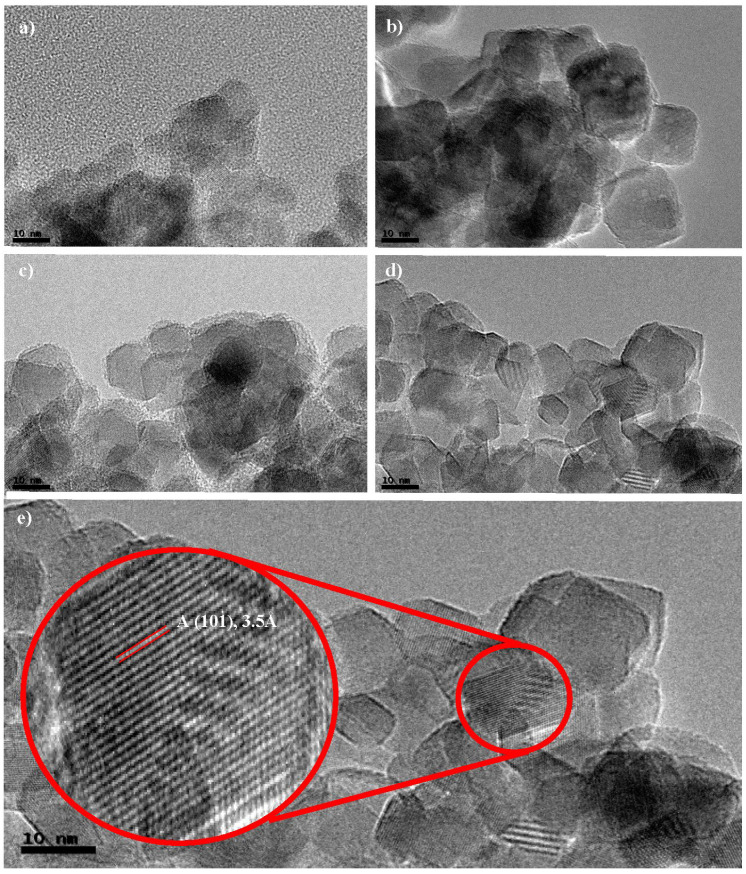
TEM images of the selected catalysts: (**a**) TDB-30, (**b**) TIP-30, (**c**) TBO-30, (**d**) TEO-30, and (**e**) TEO-30 zoomed image to observe anatase fringes.

Transmission electron micrographs (TEM) are shown in Figure 4. The images were selected from the different precursors with the same HA/P ratio as previously indicated. TEM images help to understand the morphological and crystallographic structures of the nanoparticles. It can be appreciated that TDB-30, TIP-30, TBO-30, and TEO-30 nanoparticles look similar. As in SEM images, TEM revealed agglomerations of small particles at the nanometer range. The structures observed from TEM images support the data collected in XRD results (next section), showing that anatase was the main component of the catalysts. This can be seen in Figure 4e, in the spacing patterns of the particles, which are associated with anatase [30], specifically the lattice fringes that can be associated to the crystal planes of anatase structures [31,32].

In comparison with other studies, TDB-30 and TIP-30 show a variation of single particles and agglomerations like those presented by Zhang et al. [33] and Camarillo et al. [27], apart from the lattice fringes patterns that can be observed in the images. The same can be said about the similarities between TBO-30 and TEO-30 nanoparticles in this study (Figure 4c,d) compared to the ones presented by Kinoshita et al. [20].

#### 3.2.2. XRD

**Figure 5:**
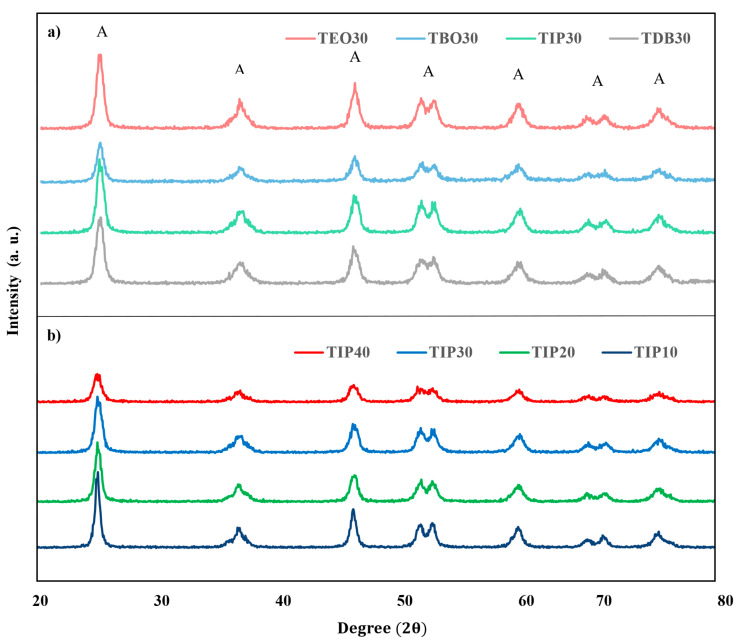
XRD diffractograms of the catalysts, indicating the anatase crystal planes: (**a**) diffractograms of TDB-30, TIP-30, TBO-30, and TEO-30; (**b**) diffractograms of the different AH/P ratios for TIP catalysts.

As part of the analyses of the physicochemical characteristics of the catalysts, XRD diffractograms were obtained. In Figure 5, we can appreciate the diffractograms of the catalysts prepared from different precursors and HA/P ratios, noticing that the peaks’ heights and definition are very similar in all of them. The distribution of the peaks indicates the presence of anatase, and the height and resolution of the peaks inform about the crystallinity of the catalysts [34,35]. Therefore, both the abundance of the anatase phase and the high and well-defined peaks are indicative of enhanced photocatalytic activity, since anatase shows a higher surface area and a higher density of active sites [12,27,36,37]. All the catalysts obtained have a crystalline structure composed of anatase. This can be appreciated in the peaks at 25.3°, 37.8°, 48.0°, 54.0°, 55.1°, 63.0°, 68.7°, 70.4°, and 75.2°. Anatase phase peaks can be indexed to the [101], [004], [200], [105], [211], [204], [116], [220], [215], [206] crystal planes, according to JCPDS card number 21-1272 [2,12]. Furthermore, there is no evidence of the main rutile peaks that are usually present at 27.5°, 36.9°, and 41.4°. The close similarity between the diffractograms and the absence of rutile could indicate that this characteristic is more driven by the temperature and pressure in the synthesis process than by the precursor and HA/P ratio used [19].

The diffractograms obtained for all the precursors exhibit similar diffraction patterns as other synthesized TiO_2_ catalysts [38,39] and as those synthesized in supercritical medium [9,27].

**Figure 6:**
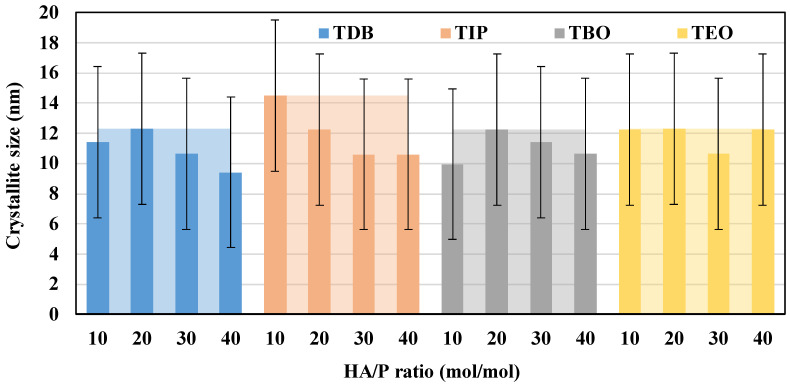
Crystallite sizes of all the catalysts synthesized. Solid color columns represent the HA/P ratio average and the clear areas behind represent the precursor maximum.

With XRD data, it is possible to calculate the percentage of the different crystal phases, as well as crystallite sizes, which are presented in Figure 6. These characteristics were obtained from the Debye–Scherrer Equation detailed elsewhere [40]. As no rutile nor brookite peaks have been detected, all the catalysts synthetized only possess anatase as crystal phase. The crystallite size data show that there is not a considerable variation between TDB, TIP, TBO, and TEO or their respective HA/P ratios, with all the catalysts crystallite sizes being around 11 nm, much smaller than the ones presented by P-25 with 20 nm.

It is also important to note that because particle size depends on reaction temperature and calcination, the slight variation presented between HA/P ratios could still be a consequence of temperature fluctuation in the reactor or in the calcination process, as it has been reported that higher temperatures tend to lead to larger particles [22,27].

Crystallite sizes displayed an improvement compared to other studies [14,19,41,42], since all catalysts prepared in this work range from 9 to 14 nm, showing a smaller crystal size than in other studies, in which they vary between 16–17 nm, and even bigger in the case of commercial catalyst P-25 (20 nm). In this work, the crystallite size is smaller than that of the catalysts obtained by Zhang et al. [40] with traditional methods, which range between 12–15 nm. This decrease in crystal size points towards an increase in the surface area, which could reduce the path length of diffuse light and transport of the charge carriers, thus enhancing the photocatalytic activity. But also, smaller crystal sizes reduce crystallinity and, consequently, the photoactivity of the catalyst [35,40].

#### 3.2.3. BET

**Figure 7:**
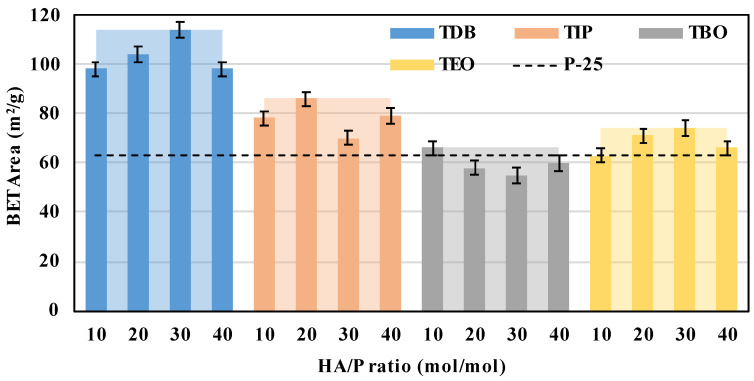
BET area of all the catalysts synthetized. Solid color columns represent the HA/P ratio average and the clear areas behind represent the precursor maximum. A commercial P-25 catalyst is also presented as a black dotted line for reference.

Another characteristic analyzed was the surface area obtained from the Brunauer–Emmett–Teller (BET) analysis, which is one of the main properties related to heterogeneous catalysis [2]. The data obtained from isothermal adsorption/desorption graphs (Figure S1) are presented in Figure 7. Taking a glance at the data, it can be noted that the precursor that generates a greater surface area is TDB, with a maximum of 114 m^2^/g, and the second highest surface area is achieved by TIP with 86 m^2^/g, meanwhile TBO and TEO produce maxima of 66 and 74 m^2^/g, respectively, being the smallest areas.

Catalysts presenting a high BET area are desired because it has been reported that the increase on this characteristic could improve the CO_2_ adsorption and photocatalytic activity [43]. According to the adsorption thermodynamics, the equilibrium uptake by a photocatalyst is a function of: (a) CO_2_ partial pressure, (b) temperature, (c) specific surface area, and (d) surface energetic. If the process is developed at constant pressure and temperature, the adsorption of CO_2_ will be controlled by certain surface properties of titania (surface area and energetics) [27]. On the one hand, a large specific surface area can accommodate more CO_2_ molecules and more photogenerated electrons. Furthermore, once reactant molecules have reached the catalyst surface, they must adsorb onto it. The adsorption capacity is linked to surface energy, which depends on the catalyst composition, among other parameters [27]. Thus, as will be seen in the next section, the presence of surface hydroxyl groups will facilitate the capture of the acidic CO_2_ and will take part in further photocatalytic reactions. For this reason, TDB and TIP could be suggested as the best precursors for catalytic or adsorption purposes.

The BET areas for every precursor present some slight variation with HA/P ratios, but again, considering the experimental error, it points out that there is no apparent direct correlation between the increase in HA/P ratios and the BET area obtained. This variation may also be related to a possible fluctuation of temperature during the catalyst’s preparation process [2].

The obtained results are in concordance with the findings of Kinoshita et al. [19], where TEO shows a smaller area than TIP and TDB. However, it defers in the case of TBO and TEO because, unlike in the present study, TEO leads to the lowest surface areas. It also must be noticed that slightly smaller surface areas are obtained compared with other studies on supercritical synthesis [14,27] or traditional synthesis [44,45,46]. This could be explained by the difference in calcination time and temperatures [19,47]. Additionally, it can be mentioned that for catalysts synthesized at relatively similar conditions, the BET areas for TDB were similar to those presented by Alonso et al. [14] and Camarillo et al. [27], but those of TIP were slightly higher than the ones obtained in this work. For TBO and TEO, the closest comparison takes place with the catalysts obtained by Kinoshita et al. [19], where BET areas were considerably smaller (around 20 m^2^/g), but those catalysts were calcinated at a higher temperature. However, we can note a trend in which TEO catalysts present smaller areas than TBO, TDB, and TIP.

#### 3.2.4. FT-IR

**Figure 8:**
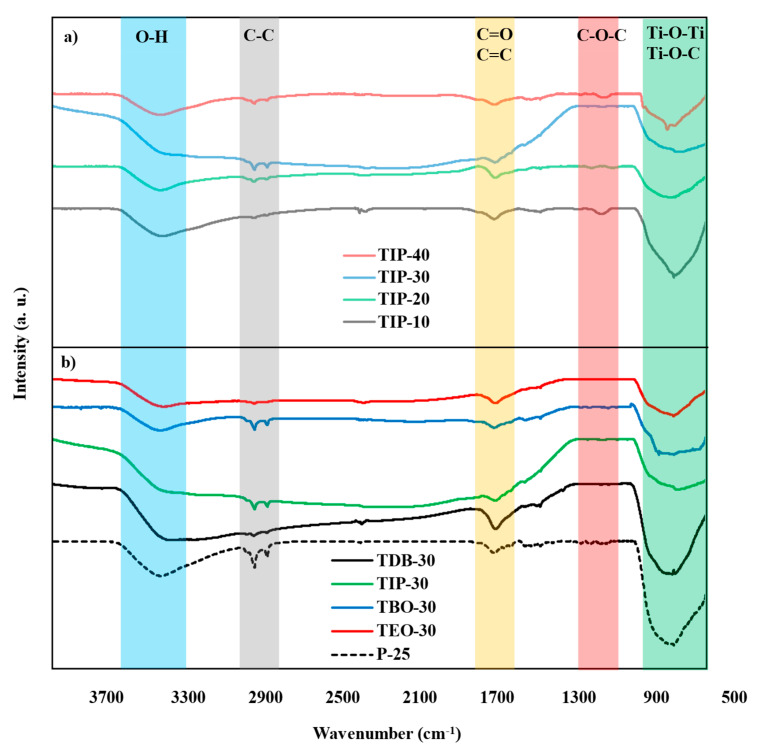
FTIR spectra of TiO_2_ catalysts: (**a**) FTIR spectra of the different TIP AH/P ratios, (**b**) FTIR spectra of TDB-30, TIP-30, TBO-30, and TEO-30. In clear transparent colors, functional groups are represented. P-25 spectrum is also presented.

FT-IR analyses were carried out to determine the surface composition and chemical bonding of the catalysts. The FTIR spectra of the selected catalysts are shown in Figure 8. As all the catalysts present a similar behavior, particularly in the group that identifies titanium bonds, it was decided to present the catalysts of the different HA/P ratios of one precursor (TIP) (Figure 8a) and the catalysts with the same HA/P ratio for the different precursors (Figure 8b). In the graphics, it can be noticed that the synthesized TiO_2_ nanoparticles exhibit a typical diversity of bands, corresponding to the functional groups expected in these types of catalysts [2]. The broad band from 3600 to 3300 cm^−1^ corresponds to the bond O–H of hydroxyl groups. Meanwhile, the C–C (CH_2_–CH_2_) skeleton stretching vibration is displayed in two small peaks around 2900 cm^−1^. The peaks around 1700 cm^−1^ and 1600 cm^−1^ belong to C=O carboxyl groups and C=C skeleton groups, respectively [2,43]. The next bands around 1200 cm^−1^ and 1000 cm^−1^ are associated to C–O of alkoxy groups and C–O of epoxy groups (C–O–C) [27,48]. Finally, the most marked peak for this catalyst can be seen between 900 and 500 cm^−1^, with these peaks being associated to the Ti–O–Ti and Ti–O–C bonds. [2]. The presence of a small peak close to 2300 cm^−1^ can be attributed either to the H-H bonds as reported by Divyasri et al. [49] or the asymmetric stretching of CO_2_ atmospheric molecules adsorbed on the surface of the catalyst reported by Chelbi et al. [28].

The FTIR spectra of the synthesized catalysts prove that TiO_2_ nanoparticles were formed in the synthesis process, evidenced by the peak present around 600 cm^−1^, being generally similar to that observed on the commercial catalyst. Also, the presence of peaks not associated to Ti has also been observed in catalysts synthesized through the traditional hydrothermal methods [21,44,50]. In principle, the presence of hydroxyl groups, carboxyl groups, and skeleton groups usually favors the photocatalytic process [37] and are a result of using ethanol as a hydrolytic agent as well as the traces of the unreacted precursors. This is reported by Camarillo et al. [27], where catalysts synthesized from isopropyl alcohol do not show other peaks, meanwhile the ones obtained from ethanol possesses these bands. O-H peaks can be linked to chemisorbed water, as well to hydroxyl groups of adsorbed water molecules [51].

#### 3.2.5. UV-vis DRS

**Figure 9:**
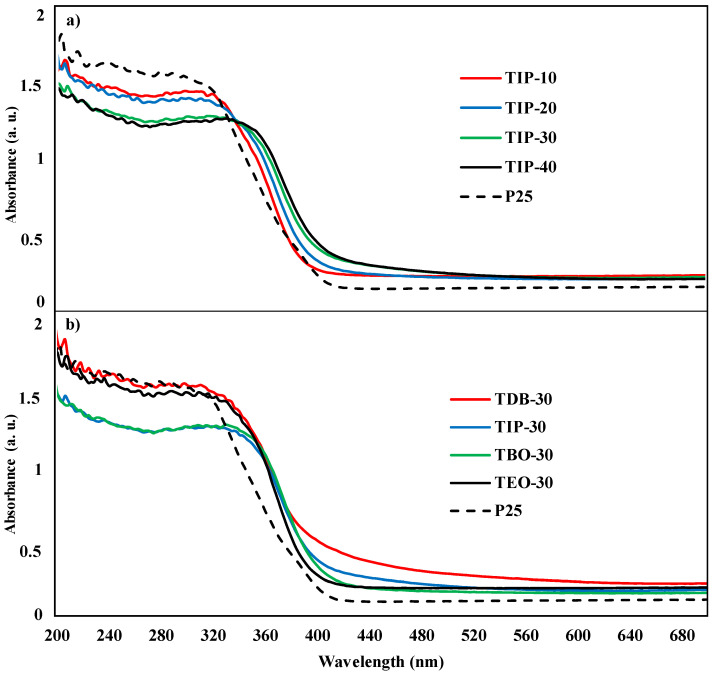
UV-vis DRS spectra of the catalysts: (**a**) UV-vis DRS spectra of the different TIP catalysts at AH/P ratios; (**b**) UV-vis DRS spectra of the selected catalysts of TDB, TIP, TBO, and TE. In both graphics, the spectrum of P-25 is presented (dotted lined).

**Figure 10:**
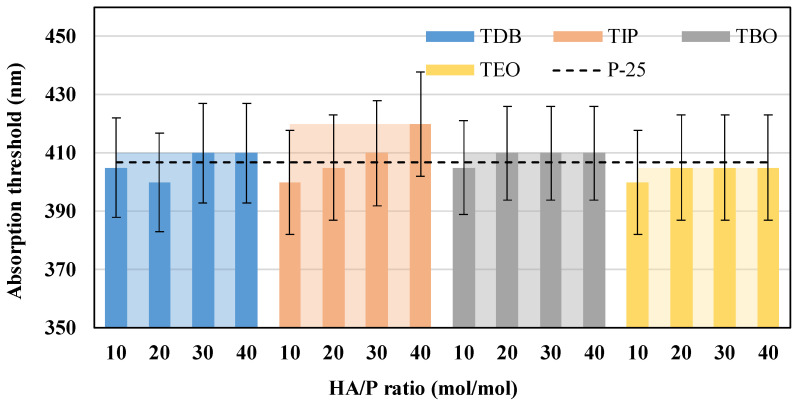
Absorption thresholds of all the catalysts and HA/P ratios, with solid color columns representing the HA/P ratio average and the clear areas behind representing the precursor maximum. A commercial P-25 catalyst is also presented as a black dotted line for reference.

**Figure 11:**
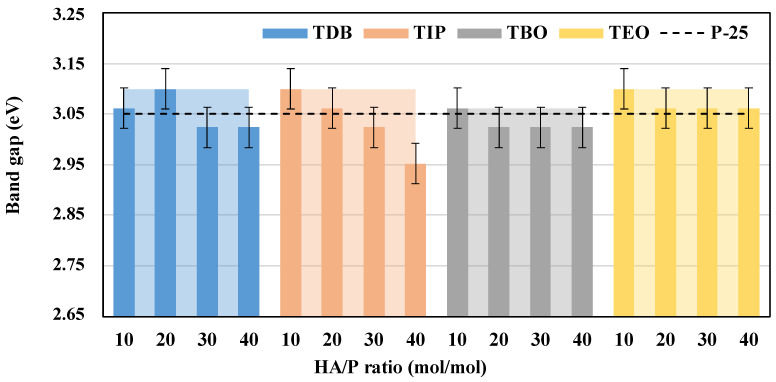
Band gap energies of all the catalysts and HA/P ratios, with solid color columns representing the HA/P ratio average and the clear areas behind representing the precursor maximum. A commercial P-25 catalyst is also presented as a black dotted line for reference.

To determine the light absorbance of the semiconductors, DRS analyses were performed on all the synthesized catalysts to study their optical characteristics. With DRS measurements, the graphics on Figure 9, Figure 10 and Figure 11 were generated. Since all slopes are in a similar range and thus hinder the identification of the catalysts, it was decided to present the catalysts of the different HA/P ratios of one precursor (TIP) in one graphic (Figure 9a) and the comparison between the same HA/P ratio (30 mol/mol) for the different precursors on the other graphic (Figure 9b). If the P-25 spectrum (dotted line) is used as a reference, it can be verified that commercial TiO_2_ typically presents intense absorption in the UV region and hardly absorbs in the visible range [2,52]. With this in mind, we can observe that all the catalysts present similar curves in the middle zone, and they all start decreasing their absorbance at around 320 nm. However, it can be noted that all the catalysts synthetized in supercritical conditions present a higher absorbance, especially in the visible region.

Subsequently, from the spectra obtained, the absorption thresholds (λ_lim_) were estimated, and the band gap energies were calculated as usual [2,27,53]. The results presented in Figure 10 show that the catalysts have absorption thresholds between 400 and 420 nm for all the precursors; however, the maximum values vary as follows: TIP > TDB = TBO > TEO. All these values are equal or higher than commercial P-25.

From these data, TIP, and TDB or TBO to a lesser extent, would be the best options for experiments where visible light is used or for modifying the catalysts to shift the absorption threshold limit towards the visible range.

Apart from TIP, which shows a slight increase on the absorption threshold as the HA/P ratio changes, starting from 400 nm to 420 nm, the remaining precursors lead to catalysts with similar threshold limits, regardless of the HA/P ratio used. For that reason, Figure 9b was selected.

These results are like the ones presented by Camarillo et al. [27], who described that the combination of ethanol and TIP produces a slightly higher absorption threshold than that of ethanol and TDB.

Regarding the band gap energies calculated, presented in Figure 11, the minima are in the range 2.95–3.06 eV for all catalysts, which was expected for this type of titanium dioxide catalyst [37]. For this parameter, both TBO and TEO are the ones that presented the least variation with respect to the HA/P ratios tested. It also should be highlighted that these values are comparable and slightly better than those reported for TiO_2_ composites produced by traditional methods [41,54,55] or TiO_2_ commercial nanoparticles [27].

#### 3.2.6. EIS

**Figure 12:**
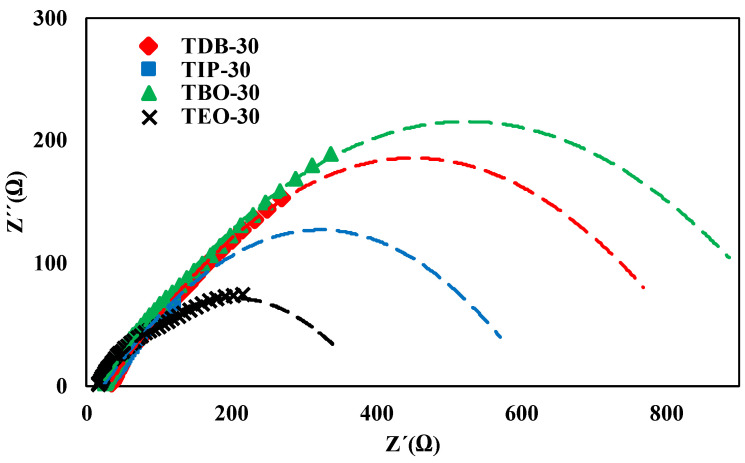
EIS Nyquist plots of selected catalysts.

Electrochemical impedance spectroscopy (EIS) is a useful technique to study the electrical behavior of photocatalytic systems [56] and the dynamics of the charges on the semiconductors [40,57,58]. It is generally used to understand the kinetics of the catalyst, measuring the rate of recombination of electron available in the conduction band [44]. The most used way to illustrate EIS is the Nyquist plots, because they are associated with the charge transfer resistance and the separation efficiency of the photogenerated e^−^/h^+^ pairs [40]. This involves comparing the imaginary impedance component (Z”) and the real impedance component (Z’) at each excitation frequency [59]. For this reason, in Figure 12, the Nyquist plots of TiO_2_ catalysts are presented. The measurements were made at room temperature for the frequency range 1 Hz and 0.1 MHz. As well as in FT-IR analysis, the catalysts selected were the ones prepared with the HA/P ratio of 30 mol/mol for TDB, TIP, TBO, and TEO.

In the Nyquist plots obtained, we can appreciate that curves appear to resemble a semicircle, as expected. The arc in the high-frequency range reveals that there is a charge transfer resistance (R_ct_) at the catalyst/electrolyte interface, which can be calculated using the Nyquist curves, with this process being described elsewhere [60]. The semicircles observed exhibit a small deviation on its origin on the real axis, showing the existence of a solution resistance (R_s_) that can be obtained from the intercept on the real axis [61,62]. These values are presented in Table 3.

The catalysts exhibit a substantial variation on their R_s_ and R_ct_ values. For example, the largest combined resistance with values of R_s_ (18.44 Ω) and R_ct_ (1069.87 Ω) is presented by the TBO-30 catalyst. Meanwhile, R_s_ and R_ct_ of the TEO-30 catalyst present a serious decrease (11.85 Ω and 378.70 Ω), which suggests that this catalyst should show improved photocatalytic activity if this feature is analyzed alone. Even though the TBO-30 catalyst presents the largest R_ct_ of the catalysts analyzed, it is not as much as the ones obtained in other TiO_2_ nanoparticles studies [37,59,63].

#### 3.2.7. Summary of Physicochemical Properties

As part of the analysis to determine the effects of precursor type and HA/P ratio on the characteristics of the catalysts, Table 4 has been prepared. As shown, they are quite similar, and it is difficult for a specific catalyst to stand out from the others. However, it can also be seen that, for any specific feature, a certain catalyst can be highlighted. In the case of production yield, the synthesis using the TIP precursor at an HA/P ratio of 40 mol/mol proves to be more the most efficient. In the case of the crystallite size, the combination of TDB and HA/P ratio of 40 mol/mol leads to the smallest value of the variable. For BET area, the combination of TDB and HA/P = 30 mol/mol causes the highest surface area, and in case of absorbance, the combination of TIP and HA/P = 40 mol/mol produces the higher absorption threshold and lower band gap energy. Finally, it should be recalled that all tested combinations of precursors and HA/P ratios led to TiO_2_ nanoparticles in the anatase crystal phase.

### 3.3. Photocatalytic Activity

Finally, the TiO_2_ nanoparticles synthesized under supercritical conditions were employed as photocatalysts for the CO_2_ photoreduction process. In all experiments, the only reaction products were CO and CH_4_, as in previous works [27].

**Figure 13:**
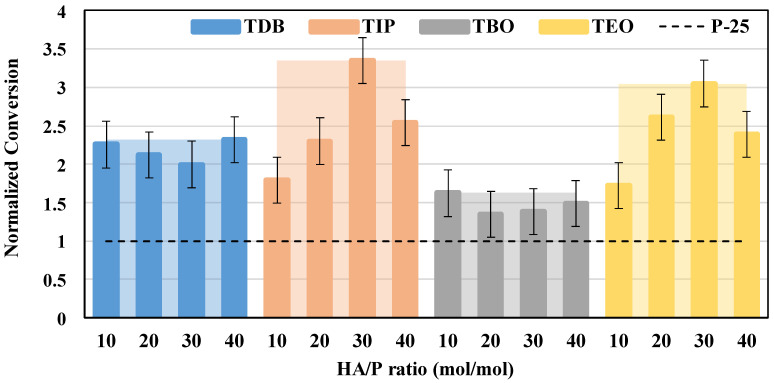
Normalized photocatalytic conversion, with solid color columns representing the HA/P ratio average and the clear areas behind representing the precursor maximum. Normalized conversion = 1 for commercial P-25 catalyst is also presented as a black dotted line for reference.

One way to compare the results obtained in this investigation with previous ones is calculating a normalized value for the CO_2_ conversion. That way it is also possible to compare the obtained conversions with those of a reference catalyst. In this study, the normalized conversions have been calculated using commercial P-25 as a reference catalyst, and they are presented in Figure 13. As shown, they are greater than unity for all our synthesized catalysts, therefore exhibiting our materials as having better CO_2_ reduction properties than P-25. Specifically, for TIP and TEO photocatalysts, higher normalized conversions are reached; they are 3.3 and 3 times higher, respectively, than those attained with P-25 for the HA/P ratio of 30 mol/mol, and an increase in CO_2_ normalized conversion is noted until this ratio is reached. TDB and TBO show the lower normalized conversions, being about 2.2 and 1.5 times higher, respectively, than the one reached with P-25. No significant effect of the HA/P ratio on CO_2_ conversion is observed with these last catalysts, the small differences observed being attributed to experimental error.

Accordingly, the catalysts that present the best conversion rates for each precursor are TIP-30, TEO-30, TDB-40, and TBO-10, therefore being the best starting points for further progress in the design of more effective TiO_2_-based catalysts.

On the other hand, it should be highlighted that data shown in Table 3 and Table 4 further support this result since these photocatalysts meet the requirements for good photocatalytic behavior, such as appropriate values of the TiO_2_ properties related to reactants adsorption, light absorption, charges generation, and transport. It should be remembered that the TIP-30 catalyst possessed the best behavior with light absorption, since this combination led to a larger absorption threshold (420 nm) and a lower band gap energy (2.95 eV). Regarding TDB-40, this combination led to smaller crystallite size (9 nm). All these results agree with those reported in a previous work [27]. In the case of TEO-30, it exhibited the lowest charge transfer and solution resistances of all catalysts tested. This good behavior could not be fully explained, but this same precursor also exhibited good photocatalytic activity in other works [20]. Nevertheless, this comparison should be made with care, since the hydrolytic agent in this case was acetic acid instead of ethanol, and the photocatalytic reaction performed was the photodegradation of methylene blue in aqueous solution instead of the photoreduction of CO_2_ in gas phase.

Another important aspect to consider is the selectivity towards the reaction products (CO and methane). Specifically, CO is an intermediate reactant, while methane represents the complete reduction of CO_2_ and has greater energy power. In this study, selectivity towards CH_4_ varies between 24% for TBO catalysts and 15% for TDB catalysts, going through 21% and 18% for TIP and TEO catalysts, respectively. All these values are higher than 2–3.5%, which is the selectivity towards CH_4_ of commercial P-25. The results for TIP and TDB are similar to those obtained in previous works [27].

To sum up, according to our results, no single characteristic seems to have a significant impact on photocatalytic reduction. However, light absorption, crystal size, surface area, and charge transfer seem to be the most influential properties on the catalyst photoactivity. This assertion is supported by the fact that the catalyst exhibiting favorable values of the photocatalytic activity also showed the highest light absorption threshold (TIP-40). A similar relation could be made for charge transfer, where TEO-30 and TIP-30, with lower charge transfer and solution resistances, produced high CO_2_ conversions. Finally, TDB-30, with the smallest crystal size, also improved CO_2_ conversion in comparison with P-25. Surface area also seems to have an influence on the photocatalytic activity, since the combinations with TBO are those with the smallest surface areas together with the poorest CO_2_ conversions.

## 4. Conclusions

The effect of the HA/P ratio is not significant for most of the precursors, either in the synthesis yield or in the catalyst properties. Only with TIP is an improvement of both production yield and physicochemical characteristics observed as the HA/P ratio increases.

In relation with the nature of precursor, the yield of the synthesis process is the parameter most influenced by this variable. Specifically, TIP is the best performing precursor, leading to a synthesis yield of 80%.

The photocatalysts producing the higher values of CO_2_ conversion are TIP-30 and TEO-30 (6.3–7 µmol·g^−1^·h^−1^), followed at a distance by TDB-40 (4.8 µmol·g^−1^·h^−1^). Regarding methane selectivity, TBO catalysts proved to be more efficient than those produced from the other precursors. Specifically, around 24% selectivity towards CH_4_ was achieved with TBO catalysts, closely followed by TIP catalysts (≈21%).

As expected, the photoreduction capacity cannot be attributed to a single physicochemical characteristic. However, it can be said that light absorption, BET surface area, crystallite size, and charge transfer properties are good indicators of the catalyst’s ability to photoreduce the CO_2_ molecules, above other characteristics such as crystallinity or particle size.

To sum up, despite not finding an overwhelming variation of the synthesis yields or catalyst properties that could directly be linked to either the type of precursor or the HA/P ratio used in the photocatalyst synthesis processes, slight differences have been found. They have been reported throughout the work and may be used as a starting point to improve the design and development of more advanced TiO_2_-based photocatalysts.

## Figures and Tables

**Table 1:** Catalyst codification.

Precursor	E/P (moL/moL)	Catalyst
Titanium diisopropoxide bis(acetylacetonate)	10	TDB-10
	20	TDB-20
	30	TDB-30
	40	TDB-40
Titanium isopropoxide	10	TIP-10
	20	TIP-20
	30	TIP-30
	40	TIP-40
Titanium tetrabutoxide	10	TBO-10
	20	TBO-20
	30	TBO-30
	40	TBO-40
Titanium ethylhexoxide	10	TEO-10
	20	TEO-20
	30	TEO-30
	40	TEO-40

**Table 2:** Formulas, structures, and hydrolysis reaction of the precursors.

Precursor and Linear Formula	Chemical Structure	Hydrolysis Reaction ^1^
TDB Ti(OC_3_H_7_)_2_(C_5_H_7_O_2_)_2_	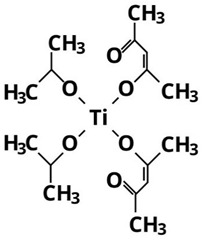	Ti(OC_3_H_7_)_2_(C_5_H_7_O_2_)_2_ + 2H_2_O → TiO_2_↓ + 2C_3_H_7_OH + 2C_5_H_7_OOH
TIP Ti(OC_3_H_7_)_4_	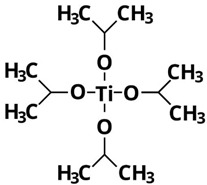	Ti(OC_3_H_7_)_4_ +2H_2_O → TiO_2_↓ + 4C_3_H_7_OH
TBO Ti[OC(CH_3_)_3_]_4_	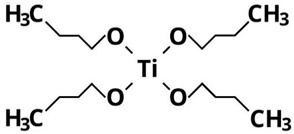	Ti[OC(CH_3_)_3_]_4_ + 2H_2_O → TiO_2_↓ + 4C(CH_3_)_3_OH
TEO Ti[OCH_2_CH(C_2_H_5_)(CH_2_)_3_CH_3_]_4_	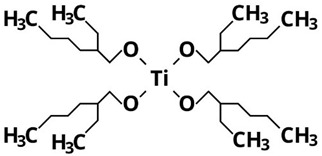	Ti[OCH_2_CH(C_2_H_5_)(CH_2_)_3_CH_3_]_4_ + 2H_2_O → TiO_2_↓ + 4CH_3_(CH_2_)_3_(C_2_H_5_)CHCH_2_OH

^1^ Water molecules for the hydrolysis reaction of the precursor result from dehydration of ethanol used as a hydrolytic agent in the solvothermal supercritical synthesis.

**Table 3:** Resistances R_s_ and R_ct_ for the selected catalysts.

Catalyst	R_s_ (Ω)	R_ct_ (Ω)
TDB-30	28.52	884.16
TIP-30	22.57	607.93
TBO-30	18.44	1069.87
TEO-30	11.85	378.70

**Table 4:** Characteristics of synthesized catalysts.

Catalyst Code	Yield (%)	Crystallite Size (nm)	BET Area (m^2^/g)	Absorption Threshold (nm)	Band Gap (eV)
TDB-10	40 ± 8	11 ± 4	98 ± 3	405 ± 20	3.06 ± 0.04
TDB-20	35 ± 8	12 ± 5	104 ± 3	400 ± 20	3.09 ± 0.04
TDB-30	37 ± 8	11 ± 4	114 ± 3	410 ± 18	3.02 ± 0.04
TDB-40	41 ± 9	9 ± 3	98 ± 3	410 ± 18	3.02 ± 0.04
TIP10	48 ± 9	14 ± 6	78 ± 3	400 ± 20	3.09 ± 0.06
TIP-20	47 ± 7	12 ± 5	86 ± 3	405 ± 20	3.06 ± 0.06
TIP-30	66 ± 7	11 ± 4	70 ± 3	410 ± 20	3.02 ± 0.06
TIP-40	80 ± 10	11 ± 4	79 ± 3	420 ± 20	2.95 ± 0.06
TBO-10	44 ± 7	10 ± 4	66 ± 3	405 ± 20	3.06 ± 0.06
TBO-20	57 ± 6	12 ± 5	58 ± 3	410 ± 18	3.02 ± 0.04
TBO-30	59 ± 7	11 ± 4	55 ± 3	410 ± 18	3.02 ± 0.04
TBO-40	52 ± 7	11 ± 4	60 ± 3	410 ± 18	3.02 ± 0.04
TEO-10	39 ± 6	12 ± 5	63 ± 3	400 ± 20	3.09 ± 0.06
TEO-20	40 ± 6	12 ± 5	71 ± 3	405 ± 20	3.06 ± 0.06
TEO-30	33 ± 9	11 ± 4	74 ± 3	405 ± 20	3.06 ± 0.06
TEO-40	39 ± 7	12 ± 5	66 ± 3	405 ± 18	3.06 ± 0.04

## Data Availability

Not applicable.

## Supplementary Materials

The following supporting information can be downloaded at: https://www.mdpi.com/article/10.3390/nano13162328/s1, Figure S1: Isothermal adsorption/desorption graphs for some catalysts: (a) TDB-30, (b) TIP-30, (c) TBO-30, (d) TEO-30.
