# Supplementary figures and images for: Impact of the Precursor on the Physicochemical Properties and Photoactivity of TiO2 Nanoparticles Produced in Supercritical CO2
Source: Nanomaterials (Basel). 2023 Aug 13;13(16):2328. doi: 10.3390/nano13162328 (PMC10459058; doi:10.3390/nano13162328)

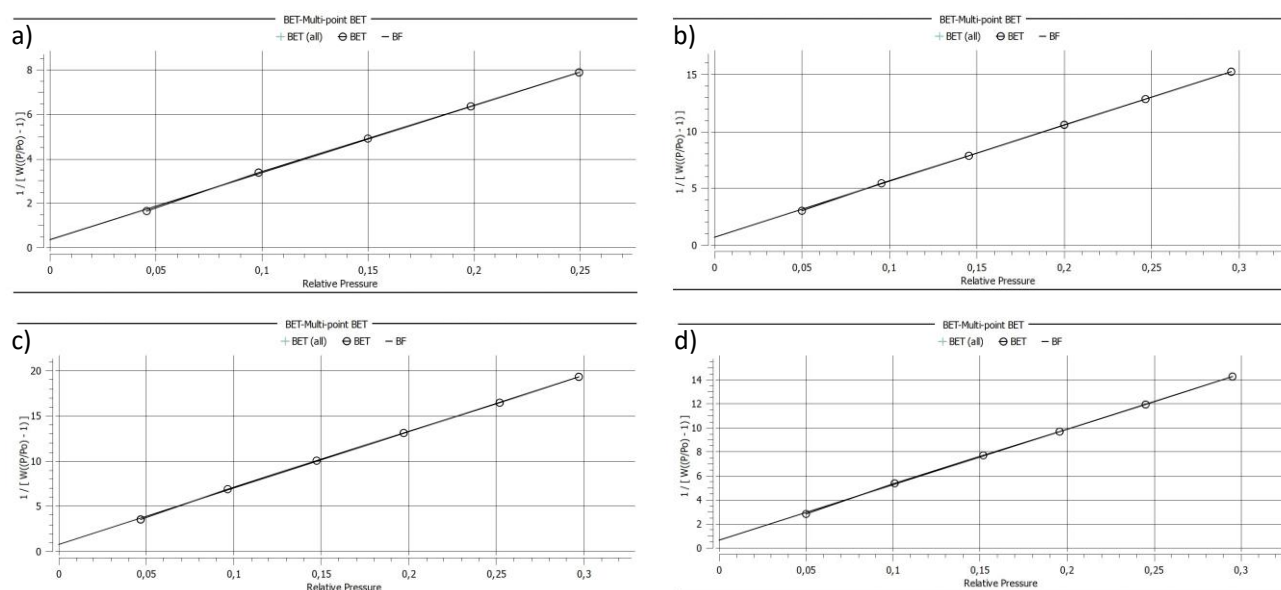

**Figure S1.** Isothermal adsorption/desorption graphs for some catalysts: a) TDB-30, b) TIP-30, c) TBO-30, d) TEO-30.

Supplement: Supplementary file 1 [file nanomaterials-13-02328-s001.zip › nanomaterials-2548549-supplementary.pdf]
